# Birth weight and premature ovarian insufficiency: a systematic review and meta-analysis

**DOI:** 10.1186/s13048-024-01357-9

**Published:** 2024-04-03

**Authors:** Chengyang Jiang, Tongqing Gao, Yuwei Wang, Wenqiang Yang, Huan Huang, Yushan Li, Xinghai Yang

**Affiliations:** 1 Department of Pediatric Surgery, Tongji Medical College, Maternal and Child Hospital of Hubei Province, Huazhong University of Science and Technology, Wuhan, 430070 Hubei China; 2 Hubei University of Chinese Medicine, Wuhan, 430065 Hubei China; 3 School of Medicine, Wuhan University of Science and Technology, Wuhan, 430065 Hubei China

**Keywords:** Low birth weight, Premature ovarian insufficiency, Birth weight

## Abstract

**Objective:**

To comprehensively evaluate the effect of low birth weight on premature ovarian insufficiency.

**Methods:**

We performed a systematic review of the literature by searching MEDLINE, EMBASE, Web of Science, Scopus, Wanfang and CNKI up to August 2023. All cohort and case-control studies that included birth weight as an exposure and premature ovarian insufficiency as an outcome were included in the analysis. Data were combined using inverse-variance weighted meta-analysis with fixed and random effects models and between-study heterogeneity evaluated. We evaluated risk of bias using the Newcastle Ottawa Scale and using Egger’s method to test publication bias. All statistical analyses were performed with the use of R software.

**Results:**

Five articles were included in the review. A total of 2,248,594 women were included, including 21,813 (1%) cases of premature ovarian insufficiency, 150,743 cases of low birth weight, and 220,703 cases of macrosomia. We found strong evidence that changed the results of the previous review that low birth weight is associated with an increased risk of premature ovarian insufficiency (OR = 1.15, 95%CI 1.09–1.22) in adulthood compared with normal birth weight. No effect of macrosomia on premature ovarian insufficiency was found.

**Conclusions:**

Our meta-analysis showed strong evidence of an association between low birth weight and premature ovarian insufficiency. We should reduce the occurrence of low birth weight by various methods to avoid the occurrence of premature ovarian insufficiency.

**Supplementary Information:**

The online version contains supplementary material available at 10.1186/s13048-024-01357-9.

## Introduce

According to the latest evidence, there are about 18 million low birth weight infants (LBW, birth weight less than 2500 g) worldwide [1]. The main causes of low birth weight are preterm birth and intrauterine growth restriction [2]. Low birth weight infants have higher early life mortality than normal birth weight infants [3]. However, there is increasing evidence that low birth weight has a significant impact not only in early life, but also throughout the life course [4].

Premature ovarian insufficiency (POI) is a disease associated with female reproduction, which means the loss of ovarian function before the age of 40 years [5]. According to the guidelines of the European Society of Human Reproduction and Embryology [6], POI was defined as two episodes of elevated FSH levels (> 25 IU/L) more than 4 weeks apart in women younger than 40 years of age with oligomenorrhea or amenorrhea for at least 4 months. The latest research results show that the global incidence of premature ovarian insufficiency is about 3.5%, of which the iatrogenic injury is about 11.2%, and the autoimmune factor is about 10.5% [7]. Of note, we found an increasing trend in the incidence of premature ovarian insufficiency [8].

At present, the etiology of premature ovarian insufficiency is not completely clear, and studies have shown that premature ovarian insufficiency is related to genetic factors, metabolic factors, and autoimmune factors [8]. However, the effect of birth weight on premature ovarian insufficiency is still unclear. We designed this meta-analysis to comprehensively examine the effect of low birth weight on premature ovarian insufficiency.

## Method

The methodology follows the MOOSE [9] statement and is explained under seven categories: search strategy, inclusion and exclusion criteria, databases, study selection, data extraction, quality assessment, and statistical analysis. (Supplementary Table 1) The protocol of this systematic review was registered in the international prospective register of systematic reviews (PROSPERO ID: CRD42023455698).

### Search strategy

The following PECO (Population, Exposure, Comparison, Outcome) elements were included in the systematic review: (1) Population: postmenopausal women; (2) Exposure: birth weight (3) Comparison: normal birth weight women; (4) Outcome: women with premature ovarian insufficiency. The search keywords were “birth weight” and “premature ovarian insufficiency “, and the deadline was August 1, 2023. The specific search formula is in the Supplementary Table 2. To identify eligible studies, a primary search was conducted in electronic databases MEDLINE, EMBASE, Scopus, Cochrane (CENTRAL), Wanfang and CNKI. In addition, we manually searched all references cited in the original studies. The primary search was performed independently by two investigators (CY J and TQ G). Discrepancies were resolved by consultation with investigators (XH Y) who were not involved in the initial procedures.

### Inclusion and exclusion criteria

Specific inclusion criteria were as follows: (1) studies on postmenopausal women; (2) studies that provide extractable data. Both cohort and case-control studies met the inclusion criteria. The following studies were excluded: (1) no control group (no normal birth weight group); (2) use of hormone therapy; (3) Women with history of polycystic ovary syndrome (PCOS) were included in the original study; (4) Women with hysterectomy or cessation of menses for other reasons were included in the original study. The authors of articles with incomplete data were contacted within the specified time limit to obtain original research data.

### Parameter definition

Low birth weight: birth weight less than 2500 g; Normal birth weight: birth weight 2500-4000 g; Macrosomia: birth weight more than 4000 g.

### Data extraction

Two investigators (CY J and TQ G) reviewed all eligible studies. The following data were extracted and recorded: (1) the first author; (2) the year of publication; (3) the country in which the study was conducted; (4) study design (case-control or cohort); (5) duration (available in cohort); (6) the total number of cases; (7) number of women diagnosed with POI (8) number of normal postmenopausal women; (9) the number of low birth weight cases in each category; (10) the number of normal birth weight cases in each category; (11) number of macrosomia cases in each category. Article screening, quality assessment, and data extraction were developed with an online software for systematic review management (Covidence.org).

### Risk of bias

Newcastle-Ottawa Quality Assessment Scale for cohort studies (NOS) was used to assess the quality of each study. It consists of eight questions composed of three axes: study selection, comparability and verification of exposure, and outcome investigated. This instrument has a classification system in which an article receives stars for each criterion met. The categories of quality classification for studies are (1) low quality—when the article receives up to 3 stars, (2) moderate quality—from 4 to 6 stars, and (3) high quality—from 7 to 9 stars [9]. (Supplementary Table 3)

### Statistical analysis

Risk factors for premature ovarian insufficiency were compared between (1) low birth weight women and normal birth weight women, and (2) normal birth weight women and macrosomia. Heterogeneity was tested using Cochrane chi-square, and I^2^ statistics were calculated. I^2^ 30–50% was considered moderate, while values > 50% were considered highly heterogeneous. Fixed and random effects models were used for data synthesis.

Associations are reported as odds ratios (OR) and their 95% confidence intervals (CI). A *p*-value of < 0.05 was considered statistically significant. Publication bias was formally tested using Egger’s test (*P*-value > 0.05 indicated no publication bias). All statistical analyses were performed with the use of R software.

## Results

### Characteristics of the studies

**Fig. 1:**
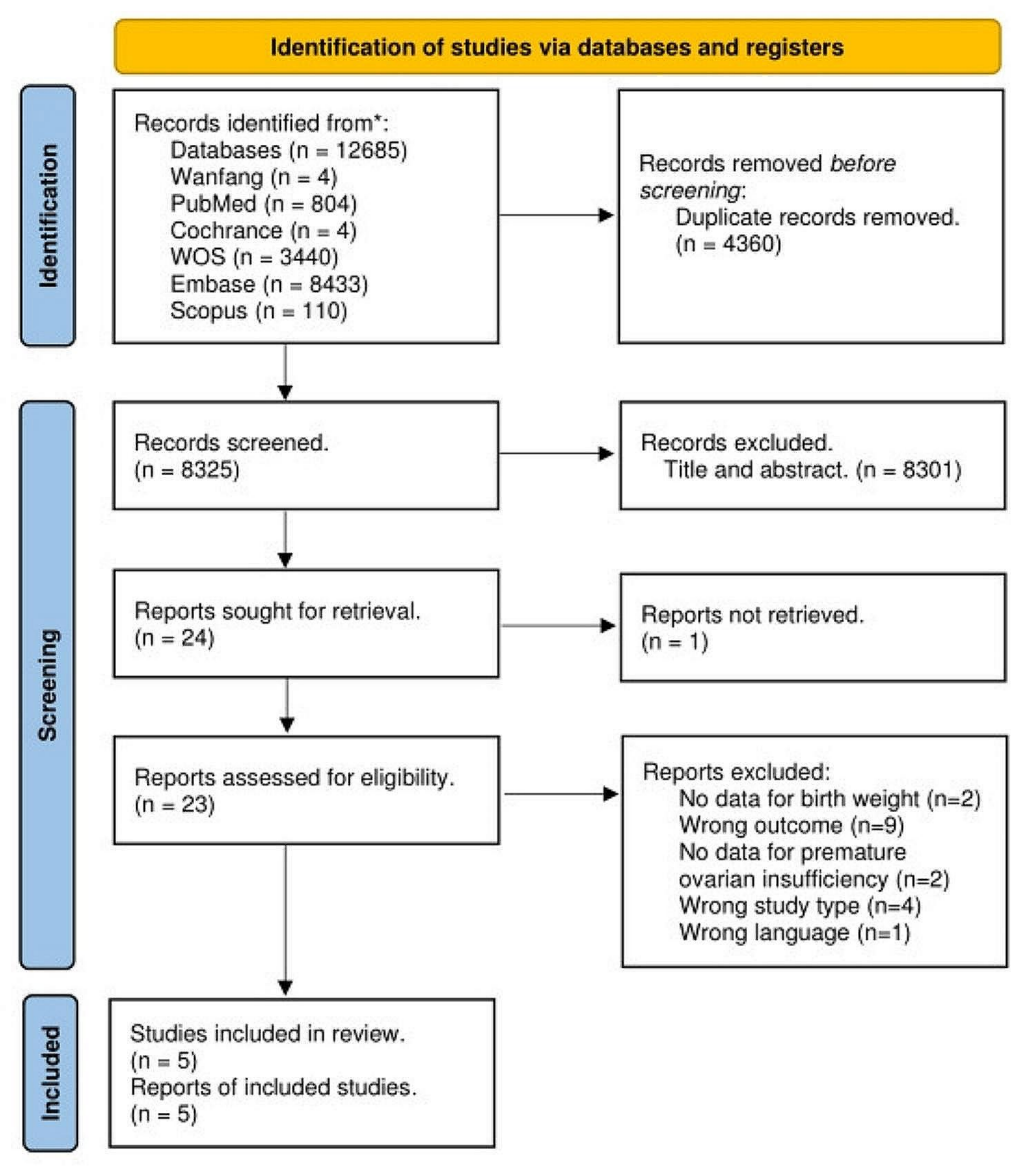
PRISMA flow diagram

After exclusion of duplicates, the initial search provided 12,685 results, of which 23 were assessed as full text after exclusion of duplicate articles. (Fig. 1) Of these, 20 articles were excluded for the following reasons: (1) Wrong outcome (*n* = 9); (2) No data for birth weight (*n* = 2); (3) No data for premature ovarian insufficiency (*n* = 2); (4) Wrong study type (*n* = 4); (5) Non-English language (*n* = 1). Five studies were included in the quantitative analysis [10–14]. The studies were published between 2010 and 2022. There were 1 case-control [10] study and 4 cohort studies [11–14]. The countries where these studies were conducted were the United Kingdom, the United States, Sweden, Norway, and the Netherlands. The number of subjects ranged from 151 to 19,892,017. A total of 22,942 women were diagnosed with POI, among whom 1248 were low birth weight infants. Descriptive information of the included study articles is summarized in Table 1.

**Table 1:** Basic information of the included studies

Author	Year	Country	Study design	Total number of subjects	follow-up period	Birth weight ascertainment method	BW reference category for adjusted estimate	NO. of POI	NOS score
Sarah E. Tom	2010	British	Cohort	3619	1958–2006	Recorded from medical records	< 2.5;2.5–2.9;3.0-3.49;3.5–3.9;≥4.0	215	8
Christine R Langton	2022	US states	Cohort	19892017	1989–2017	Recorded from medical records	< 2.5;2.5–2.9;3.0-3.49;3.5–3.9;≥4.0	2135	7
Gunilla Sydsjö	2020	Sweden	Cohort	1033878	1973–2012	Recorded from medical records	< 1.5;1.5–2.5;≥2.5	18627	9
S. Sadrzadeh	2017	Netherlands	Case-control	151	NA	self-report	< 1.5;1.5-2.0;2.0-2.5;2.5-4.0;≥4.0	59	4
Elisabeth K Bjelland	2020	Norway	Cohort	164608	1936–2014	self-report	< 2.5;2.5–2.9;3.0-3.49;3.5–3.9;4.0-4.49;≥4.5	1906	7

NA: Case-control

### Risk of bias

The quality assessment of the included studies is shown in Supplementary Table 2. All cohort study is rated “low risk of bias”. One case-control study was also rated as having a “moderate risk of bias”.

### LBW and POI

**Fig. 2:**
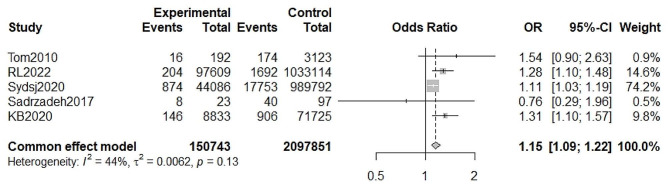
Meta-analysis for the association between of low birth weight and premature ovarian insufficiency

**Fig. 3:**
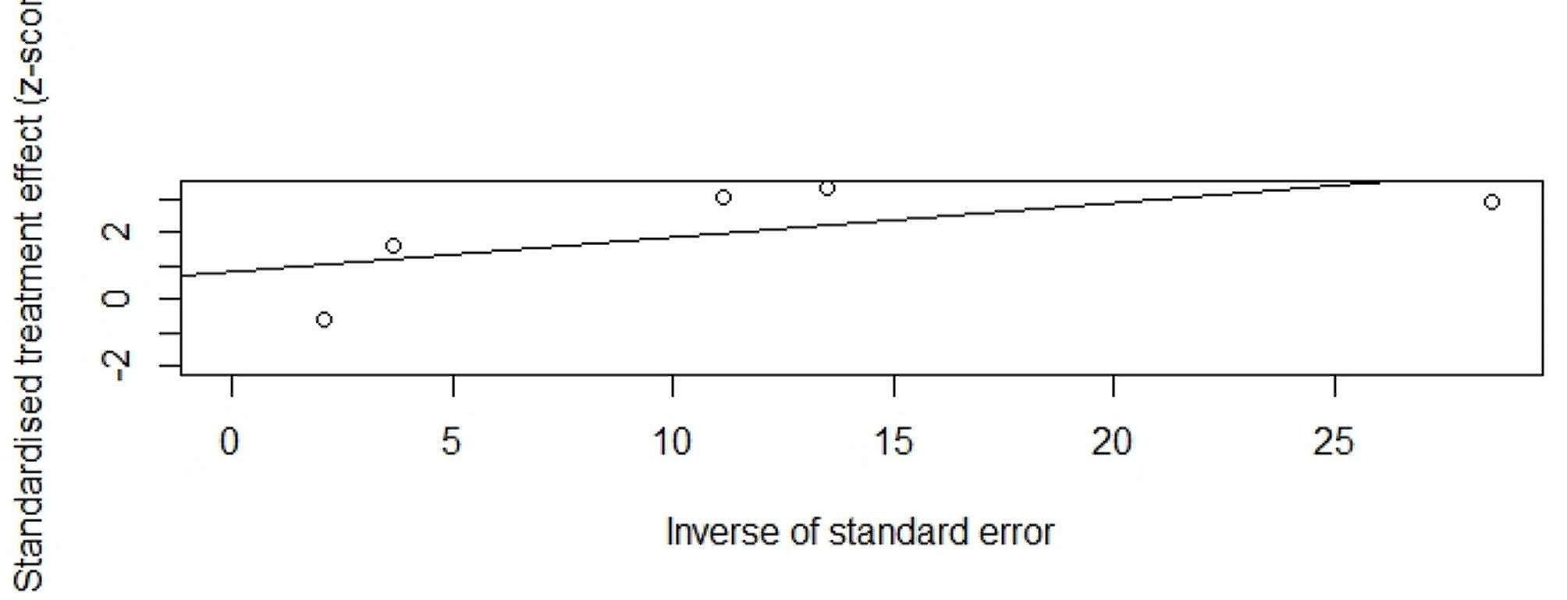
Egger’s test for BLW and POI

A total of five studies provided available data for the calculation of odds ratios for premature ovarian insufficiency in low birth weight infants compared with normal birth weight infants. Meta-analysis using a fixed effect model showed a significant association between low birth weight and premature ovarian insufficiency, with a pooled OR = 1.15 (95%CI, 1.09–1.22, I^2^ = 44.3%, P _heterogeneity_ >0.05) (Fig. 2). No significant publication bias was found by Egger’s test (t = 0.87, *P* = 0.4501) (Fig. 3). The random-effects model was used to reanalyze the results, with OR of 1.21 (95%CI, 1.08–1.35, I^2^ = 44.3%, P _heterogeneity_ >0.05). This is similar to our previous results.

### Macrosomia and POI

**Fig. 4:**
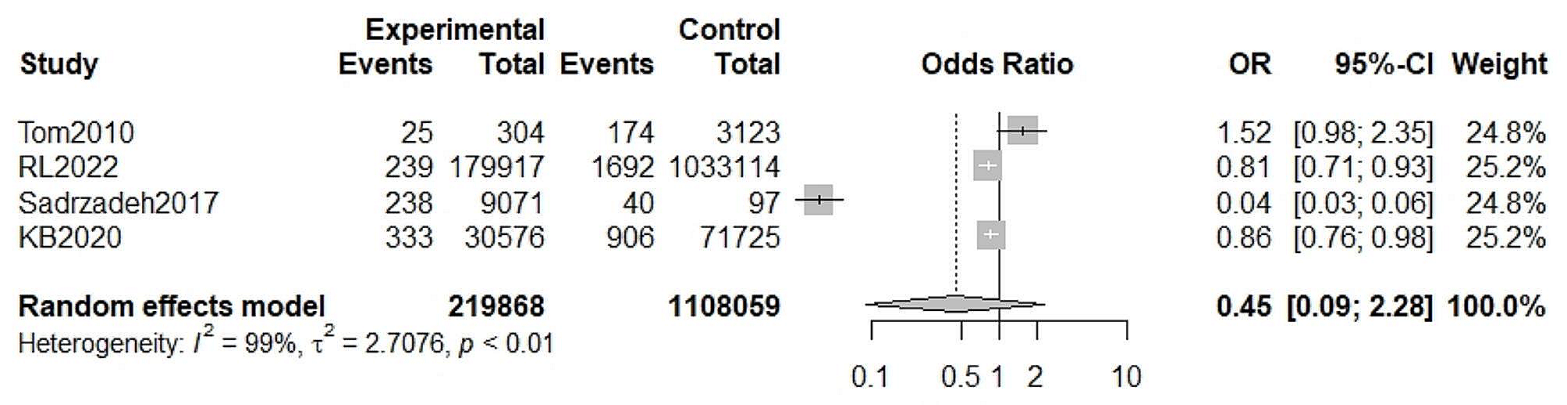
Meta-analysis for the association between of macrosomia and premature ovarian insufficiency

**Fig. 5:**
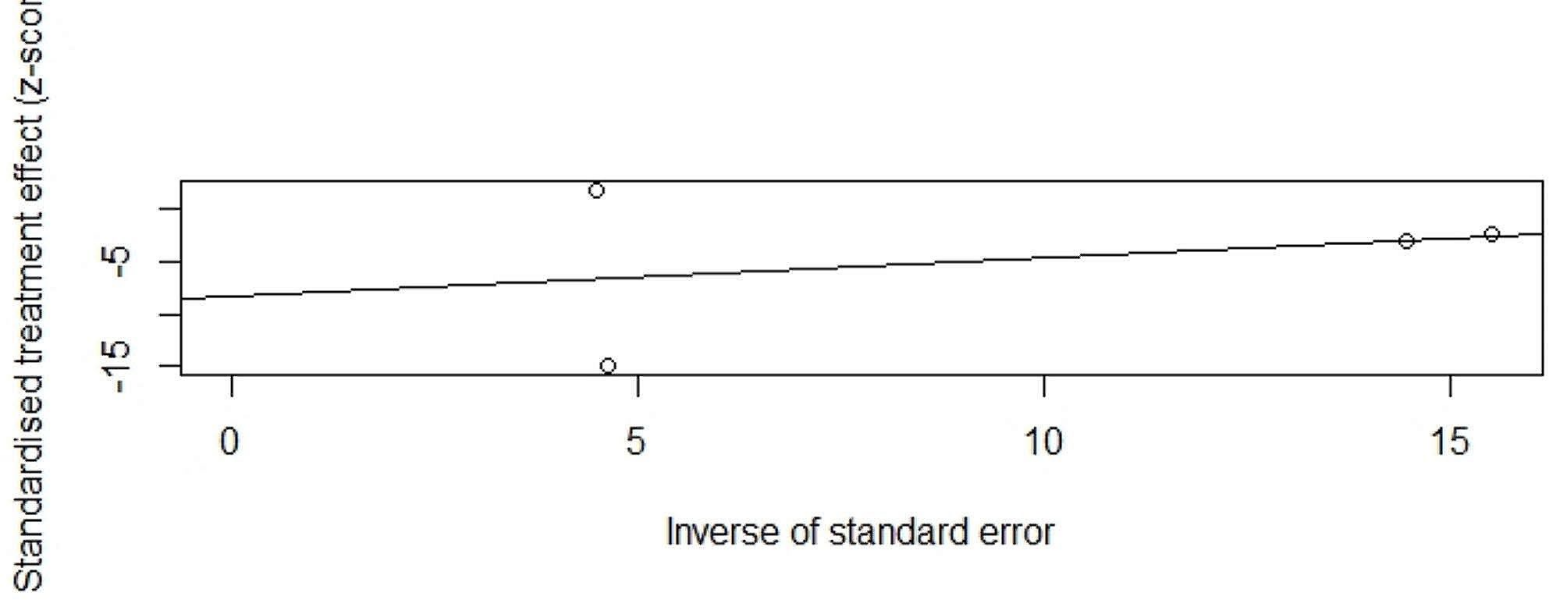
Egger’s test for Macrosomia and POI

A total of four studies provided available data for the calculation of odds ratios for premature ovarian insufficiency in macrosomia versus normal birth weight infants. Using a random effects model, the meta-analysis showed no significant association between macrosomia and premature ovarian insufficiency, with a pooled OR = 0.45(95%CI, 0.09–2.28, I^2^ = 99%, P _heterogeneity_ <0.05) (Fig. 4). No significant publication bias was found by Egger’s test(t = -0.91, *P* = 0.4577). (Fig. 5).

### Sensitivity analysis

Among low-birth-weight infants, the results changed when we used the one-by-one method to exclude the Sydsjö [10] study, but we found that the effect sizes increased, and the differences became more significant. Excluding other studies, we did not find significant changes in the results. We obtained similar results to fixed effects after reanalysis using a random effects model. We consider the results to be robust. In the comparison of macrosomia and normal birth weight infants, excluding any of the literatures did not change the results significantly.

## Discussion

In our combined analysis, we found that low-birth-weight women had a 15% higher risk of premature ovarian insufficiency in adulthood than did normal-birth-weight women. However, we did not find a protective effect of higher birth weight on premature ovarian insufficiency as weight increased.

In contrast to a review a decade ago, it was generally accepted that low birth weight had no significant effect on premature ovarian insufficiency [15]. Due to the results of large cohort studies in recent years [10–14], we came to the opposite conclusion. After incorporating the large sample cohort studies in recent years, we used standardized methods to synthesize the results. To the best of our knowledge, this study is currently the first meta-analysis of low birth weight for premature ovarian insufficiency.

Although they found no effect of birth weight on age at menopause between monozygotic and dizygotic twins in a previous study of twins, they also showed that there was a significant difference in birth weight between twins with premature menopause (age < 35 years) [16]. Also looking at famine-era populations, each kilogram increase in birth weight was associated with a 22% reduction in the hazard of early menopause, and adjustment for smoking did not change this estimate [17]. 

We found a significant association between low birth weight and the occurrence of premature ovarian insufficiency. However, as birth weight increased, there was no significant protective effect. We included a large sample of cohort studies and used standardized methods to synthesize the study results and reach reliable conclusions.

It has been reported in previous articles that low birth weight is not only associated with a longer length of hospital stay at birth and cost of production, but also has been shown to have a significant effect on all-cause mortality at the end of life [18]. In addition, many late-onset diseases have been confirmed to be associated with low birth weight, such as diabetes, asthma, leukemia, neuroblastoma, and hypertension [19–24].

Some clinical and experimental studies have shown that early intrauterine dysplasia can lead to the decline of ovarian follicle reserve, the change of ovulation rate [25] and the change of menarche age [26]. Studies have shown that oligoovulation and anovulation are more common in low birth weight and small for gestational age infants during puberty than in normal children [27]. It has also been suggested that a lower birth weight is associated with an earlier onset of menarche in female children [28], which may also lead to an earlier cessation of ovulation.

Morphological studies have also shown that the development of various organs of the body in low birth weight fetuses has changed compared with normal birth weight infants [29]. It has been confirmed that female infants with anatomically low birth weight have a lower proportion of ovarian volume than normal female infants [30]. The endocrine overproduction of FSH occurs in low-birth-weight women, and FSH and insulin are thought to be key hormones that influence spontaneous ovulation [31].

Animal studies have also shown that low birth weight mice exhibit different reproductive cycles from normal mice due to leptin, estrogen, and insulin resistance. Meanwhile, the number of corpus luteum and small follicles in the ovaries of LBW mice was significantly reduced, while the number of large cystic structures indicating anovulation was increased [32].

Some of the causes of low birth weight are influenced by maternal nutrition during pregnancy [33]. Interestingly, the Dutch study showed that women with low birth weight due to starvation also had offspring with low birth weight [34]. This may lead to intergenerational transmission of ovarian developmental and endocrine abnormalities associated with low birth weight. We need to take more measures to improve the nutrition of pregnant women and other measures to prevent intergenerational transmission caused by nutritional conditions [35].

## Conclusion and implications for clinical practice and future research

We used standardized procedures to comprehensively assess the risk of low birth weight and premature ovarian insufficiency, including multiple large sample cohort studies to minimize bias, and finally concluded that low birth weight was a significant risk factor for premature ovarian insufficiency. Although we have not been able to determine the effect of birth weight on premature ovarian insufficiency with a U-shaped shape due to limitations of the original study, a comprehensive approach is needed to reduce the risk of low birth weight.

Although we have established the effect of low birth weight on premature ovarian insufficiency, we have not known whether effective measures exist to improve the outcomes of premature ovarian insufficiency in early life. Future experiments with larger samples may address this issue.

## Limitations of our study

Although we tried to minimize bias, birth weight was self-reported in some studies, which inevitably introduces recall bias. At the same time, limited by the characteristics of observational research, we cannot completely rule out all potential confounding factors, which still needs further reliable experiments with large samples. At the same time, the number of studies included in this Meta-analysis was small, and more studies may be needed to verify the results in the future.

### Electronic supplementary material

Below is the link to the electronic supplementary material.

Supplementary Material 1

## References

[1] Lee AC, Katz J, Blencowe H (2013). National and regional estimates of term and preterm babies born small for gestational age in 138 low-income and middle-income countries in 2010. Lancet Global Health.

[2] Kramer MS (1987). Intrauterine growth and gestational duration determinants. Pediatrics.

[3] Lee AC, Kozuki N, Cousens S (2017). Estimates of burden and consequences of infants born small for gestational age in low and middle income countries with INTERGROWTH-21(St) standard: analysis of CHERG datasets. BMJ.

[4] Sania A, Sudfeld CR, Danaei G et al. Early life risk factors of motor, cognitive and language development: a pooled analysis of studies from low/middle-income countries. BMJ Open 2019;9:e026449. 10.1136/bmjopen-2018-026449 PMC6797384 31585969

[5] Webber L, Davies M, Anderson R, Bartlett J, Braat D, Cartwright B, Cifkova R, de Keizer-Schrama M, Hogervorst S, Janse E (2016). ESHRE Guideline: management of women with premature ovarian insufficiency. Hum Reprod.

[6] ESHRE, POI, and Guideline Development Group. Management of women with premature ovarian insufficiency. Guideline Eur Soc Hum Reprod Embryol (2015):56–7.

[7] Li M, Zhu Y, Wei J, Chen L, Chen S, Lai D (2023). The global prevalence of premature ovarian insufficiency: a systematic review and meta-analysis. Climacteric.

[8] Chon SJ, Umair Z, Yoon MS (2021). Premature ovarian insufficiency: past, Present, and Future. Front Cell Dev Biol.

[9] Brooke BS, Schwartz TA, Pawlik TM. MOOSE Reporting Guidelines for Meta-analyses of Observational Studies. JAMA Surg. 2021;156(8):787–788. 10.1001/jamasurg.2021.0522. PMID: 33825847. 10.1001/jamasurg.2021.0522 33825847

[10] Sadrzadeh S, Painter RC, van Kasteren YM, Braat DD, Lambalk CB (2017). Premature ovarian insufficiency and perinatal parameters: a retrospective case-control study. Maturitas.

[11] Sydsjö G, Bladh M, Rindeborn K, Hammar M, Rodriguez-Martinez H, Nedstrand E (2020). Being born preterm or with low weight implies a risk of infertility and premature loss of ovarian function; a national register study. Ups J Med Sci.

[12] Tom SE, Cooper R, Kuh D, Guralnik JM, Hardy R, Power C (2010). Fetal environment and early age at natural menopause in a British birth cohort study. Hum Reprod.

[13] Bjelland EK, Gran JM, Hofvind S, Eskild A (2020). The association of birthweight with age at natural menopause: a population study of women in Norway. Int J Epidemiol.

[14] Langton CR, Whitcomb BW, Purdue-Smithe AC, Sievert LL, Hankinson SE, Manson JE, Rosner BA, Bertone-Johnson ER (2022). Association of in Utero exposures with risk of early natural menopause. Am J Epidemiol.

[15] Sadrzadeh S, Verschuuren M, Schoonmade LJ, Lambalk CB, Painter RC (2018). The effect of adverse intrauterine conditions, early childhood growth and famine exposure on age at menopause: a systematic review. J Dev Orig Health Dis.

[16] Susan A. Treloar and others, birth weight and age at menopause in Australian female twin pairs: exploration of the fetal origin hypothesis, Human Reproduction, 2000;15(1):55–9. 10.1093/humrep/15.1.55. 10.1093/humrep/15.1.55 10611188

[17] Yarde F, Broekmans FJ, van der Pal-de Bruin KM, Schönbeck Y, te Velde ER, Stein AD, Lumey LH (2013). Prenatal famine, birthweight, reproductive performance and age at menopause: the Dutch hunger winter families study. Hum Reprod.

[18] Risnes KR, Vatten LJ, Baker JL (2011). Birthweight and mortality in adulthood: a systematic review and meta-analysis. Int J Epidemiol.

[19] Belbasis L, Savvidou MD, Kanu C (2016). Birth weight in relation to health and disease in later life: an umbrella review of systematic reviews and meta-analyses. BMC Med.

[20] Martín-Calvo N, Goni L, Tur JA, Martínez JA (2022). Low birth weight and small for gestational age are associated with complications of childhood and adolescence obesity: systematic review and meta-analysis. Obes Rev.

[21] ebrahtu TF, Feltbower RG, Greenwood DC, Parslow RC (2015). Birth weight and childhood wheezing disorders: a systematic review and meta-analysis. J Epidemiol Community Health.

[22] Caughey RW, Michels KB (2009). Birth weight and childhood leukemia: a meta-analysis and review of the current evidence. Int J Cancer.

[23] Thomas Harder and others. Birth weight and risk of neuroblastoma: a meta-analysis. Int J Epidemiol. June 2010;39(3):746–56. 10.1093/ije/dyq040 20236985

[24] Mu M, Wang SF, Sheng J, Zhao Y, Li HZ, Hu CL, Tao FB (2012). Birth weight and subsequent blood pressure: a meta-analysis. Arch Cardiovasc Dis.

[25] Sloboda DM, Howie GJ, Pleasants A, Gluckman PD, Vickers MH (2009). Pre- and postnatal Nutritional histories Influence Reproductive maturation and ovarian function in the rat. PLoS ONE.

[26] Gardner DS, Ozanne SE, Sinclair KD (2009). Effect of the early-life Nutritional Environment on Fecundity and Fertility of mammals. Philos Trans R Soc Lond B Biol Sci.

[27] Ibáñez L, Potau N, Ferrer A, Rodriguez-Hierro F, Marcos MV, de Zegher F (2002). Reduced ovulation rate in adolescent girls born small for gestational age. J Clin Endocrinol Metab.

[28] Wang L, Xu F, Zhang Q, Chen J, Zhou Q, Sun C (2023). Causal relationships between birth weight, childhood obesity and age at menarche: a two-sample mendelian randomization analysis. Clin Endocrinol (Oxf).

[29] Fowden AL, Giussani DA, Forhead AJ (2005). Endocrine and metabolic programming during intrauterine development. Early Hum Dev.

[30] Bruin JP, Dorland M, Bruinse HW, Spliet W, Nikkels PG, Te Velde ER (1998). Fetal growth retardation as a cause of impaired ovarian development. Early Hum Dev.

[31] Ibáñez L, Valls C, Cols M, Ferrer A, Marcos MV, De Zegher F (2002). Hypersecretion of FSH in infant boys and girls born small for gestational age. J Clin Endocrinol Metab.

[32] Khorram O, Keen-Rinehart E, Chuang TD, Ross MG, Desai M (2015). Maternal undernutrition induces premature reproductive senescence in adult female rat offspring. Fertil Steril.

[33] Stephenson T, Symonds ME (2002). Maternal nutrition as a determinant of birth weight. Arch Dis Child Fetal Neonatal Ed.

[34] Zambrano E, Martinez-Samayoa PM, Bautista CJ, Deas M, Guillen L, Rodriguez-Gonzalez GL, Guzman C, Larrea F, Nathanielsz P (2005). Sex differences in transgenerational alterations of growth and metabolism in progeny (F2) of female offspring (F1) of rats fed a low protein diet during pregnancy and lactation. J Physiol.

[35] Ramakrishnan U. Nutrition and low birth weight: from research to practice. Am J Clin Nutr. 2004;79(1):17–21. 10.1093/ajcn/79.1.17 PMID: 14684392. 10.1093/ajcn/79.1.17 14684392

