# Supplementary material for: Birth weight and premature ovarian insufficiency: a systematic review and meta-analysis
Source: J Ovarian Res. 2024 Apr 3;17:74. doi: 10.1186/s13048-024-01357-9 (PMC10988833; doi:10.1186/s13048-024-01357-9)
Supplement: Supplementary file 1 — Supplementary Material 1 [file 13048_2024_1357_MOESM1_ESM.docx]

**Supplementary table 1.** MOOSE (Meta-analyses OF Observational Studies in Epidemiology) checklist.

| **Item No** | **Recommendation** | **Reported on page** |
| --- | --- | --- |
| **Reporting of background should include** | | |
| 1 | Problem definition | 2 |
| 2 | Hypothesis statement | 2 |
| 3 | Description of study outcome(s) | 2 |
| 4 | Type of exposure or intervention used | 2 |
| 5 | Type of study designs used | 2 |
| 6 | Study population | 2 |
| **Reporting of search strategy should include** | | |
| 7 | Qualifications of searchers (eg, librarians and investigators) | 5 |
| 8 | Search strategy, including time period included in the synthesis and key words | 5 |
| 9 | Effort to include all available studies, including contact with authors | 5 |
| 10 | Databases and registries searched | 5 |
| 11 | Search software used, name and version, including special features used (eg, explosion) | 5 |
| 12 | Use of hand searching (eg, reference lists of obtained articles) | 5 |
| 13 | List of citations located and those excluded, including justification | Figure 1, Table S4 |
| 14 | Method of addressing articles published in languages other than English | 5 |
| 15 | Method of handling abstracts and unpublished studies | N/A |
| 16 | Description of any contact with authors | 5 |
| **Reporting of methods should include** | | |
| 17 | Description of relevance or appropriateness of studies assembled for assessing the hypothesis to be tested | 4 |
| 18 | Rationale for the selection and coding of data (eg, sound clinical principles or convenience) | 4 |
| 19 | Documentation of how data were classified and coded (eg, multiple raters, blinding and interrater reliability) | 5 |
| 20 | Assessment of confounding (eg, comparability of cases and controls in studies where appropriate) | 5 |
| 21 | Assessment of study quality, including blinding of quality assessors, stratification or regression on possible predictors of study results | 5 |
| 22 | Assessment of heterogeneity | 7 |
| 23 | Description of statistical methods (eg, complete description of fixed or random effects models, justification of whether the chosen models account for predictors of study results, dose-response models, or cumulative meta-analysis) in sufficient detail to be replicated | 6 |
| 24 | Provision of appropriate tables and graphics | Figures 1-3, Table 1 |
| **Reporting of results should include** | | |
| 25 | Graphic summarizing individual study estimates and overall estimate | Table 1 |
| 26 | Table giving descriptive information for each study included | Table 1 |
| 27 | Results of sensitivity testing (eg, subgroup analysis) | 8 |
| 28 | Indication of statistical uncertainty of findings | Figures 2,3,4 |
| **Reporting of discussion should include** | | |
| 29 | Quantitative assessment of bias (eg, publication bias) | Table S2 |
| 30 | Justification for exclusion (eg, exclusion of non-English language citations) | Table S4 |
| 31 | Assessment of quality of included studies | Table S3 |
| **Reporting of conclusions should include** | | |
| 32 | Consideration of alternative explanations for observed results | 12 |
| 33 | Generalization of the conclusions (ie, appropriate for the data presented and within the domain of the literature review) | 10 |
| 34 | Guidelines for future research | 10 |
| 35 | Disclosure of funding source | 11 |

**Supplementary table 2.** The search processes.

PUBMED (Searched on: 1 August 2022)

| Search number | Query | Results | Time |
| --- | --- | --- | --- |
| #1 | ((((Birth Weight[MeSH Terms]) OR (Infant, Extremely Low Birth Weight[MeSH Terms])) OR (Infant, Very Low Birth Weight[MeSH Terms])) OR (Infant, Low Birth Weight[MeSH Terms])) OR (Infant, Large for Gestational Age[MeSH Terms]) | [78,112](https://pubmed.ncbi.nlm.nih.gov/?term=%28%28%28%28Birth+Weight%5BMeSH+Terms%5D%29+OR+%28Infant%2C+Extremely+Low+Birth+Weight%5BMeSH+Terms%5D%29%29+OR+%28Infant%2C+Very+Low+Birth+Weight%5BMeSH+Terms%5D%29%29+OR+%28Infant%2C+Low+Birth+Weight%5BMeSH+Terms%5D%29%29+OR+%28Infant%2C+Large+for+Gestational+Age%5BMeSH+Terms%5D%29&sort=) | 01:41:38 |
| #2 | (((((Birth Weight) OR (Extremely Low Birth Weight)) OR (Very Low Birth Weight)) OR (Low Birth Weight)) OR (Large Gestational Age)) OR (birth characteristics) | [171,605](https://pubmed.ncbi.nlm.nih.gov/?term=%28%28%28%28%28Birth+Weight%29+OR+%28Extremely+Low+Birth+Weight%29%29+OR+%28Very+Low+Birth+Weight%29%29+OR+%28Low+Birth+Weight%29%29+OR+%28Large+Gestational+Age%29%29+OR+%28birth+characteristics%29&sort=) | 01:43:24 |
| #3 | #1OR#2 | [171,605](https://pubmed.ncbi.nlm.nih.gov/?term=%28%28%28%28%28%28Birth+Weight%29+OR+%28Extremely+Low+Birth+Weight%29%29+OR+%28Very+Low+Birth+Weight%29%29+OR+%28Low+Birth+Weight%29%29+OR+%28Large+Gestational+Age%29%29+OR+%28birth+characteristics%29%29+OR+%28%28%28%28%28Birth+Weight%5BMeSH+Terms%5D%29+OR+%28Infant%2C+Extremely+Low+Birth+Weight%5BMeSH+Terms%5D%29%29+OR+%28Infant%2C+Very+Low+Birth+Weight%5BMeSH+Terms%5D%29%29+OR+%28Infant%2C+Low+Birth+Weight%5BMeSH+Terms%5D%29%29+OR+%28Infant%2C+Large+for+Gestational+Age%5BMeSH+Terms%5D%29%29&sort=) | 01:43:41 |
| #4 | Primary Ovarian Insufficiency[MeSH Terms] | [3,469](https://pubmed.ncbi.nlm.nih.gov/?term=Primary+Ovarian+Insufficiency%5BMeSH+Terms%5D&sort=) | 01:37:25 |
| #5 | (((Primary Ovarian Failure) OR (Premature Ovarian Failure)) OR (Premature Ovarian Insufficiency)) OR (menopause) | [100,040](https://pubmed.ncbi.nlm.nih.gov/?term=%28%28%28Primary+Ovarian+Failure%29+OR+%28Premature+Ovarian+Failure%29%29+OR+%28Premature+Ovarian+Insufficiency%29%29+OR+%28menopause%29&sort=) | 01:38:14 |
| #6 | #4OR#5 | [100,040](https://pubmed.ncbi.nlm.nih.gov/?term=%28%28%28%28Primary+Ovarian+Failure%29+OR+%28Premature+Ovarian+Failure%29%29+OR+%28Premature+Ovarian+Insufficiency%29%29+OR+%28menopause%29%29+OR+%28Primary+Ovarian+Insufficiency%5BMeSH+Terms%5D%29&sort=) | 01:38:36 |
| #7 | #3AND#6 | [804](https://pubmed.ncbi.nlm.nih.gov/?term=%28%28%28%28%28%28%28Birth+Weight%29+OR+%28Extremely+Low+Birth+Weight%29%29+OR+%28Very+Low+Birth+Weight%29%29+OR+%28Low+Birth+Weight%29%29+OR+%28Large+Gestational+Age%29%29+OR+%28birth+characteristics%29%29+OR+%28%28%28%28%28Birth+Weight%5BMeSH+Terms%5D%29+OR+%28Infant%2C+Extremely+Low+Birth+Weight%5BMeSH+Terms%5D%29%29+OR+%28Infant%2C+Very+Low+Birth+Weight%5BMeSH+Terms%5D%29%29+OR+%28Infant%2C+Low+Birth+Weight%5BMeSH+Terms%5D%29%29+OR+%28Infant%2C+Large+for+Gestational+Age%5BMeSH+Terms%5D%29%29%29+AND+%28%28%28%28%28Primary+Ovarian+Failure%29+OR+%28Premature+Ovarian+Failure%29%29+OR+%28Premature+Ovarian+Insufficiency%29%29+OR+%28menopause%29%29+OR+%28Primary+Ovarian+Insufficiency%5BMeSH+Terms%5D%29%29&sort=) | 01:45:17 |

**Supplementary table 3.** Quality assessment (Newcastle-Ottawa Scale [NOS]).

| Studies | Representativeness of the exposed cohort | Selection of non-exposed cohort | Ascertainment of exposure factor | Demonstration that outcome of interest was not present at start of study | Comparability of cohorts on the basis of the design or analysis (★★) | Evaluation of outcome | Was follow-up long enough for outcomes to occur | Adequacy of follow-up of cohorts | Quality Scores |
| --- | --- | --- | --- | --- | --- | --- | --- | --- | --- |
| Sarah E. Tom 2010 | ★ | ★ | ★ | ★ | ★ | ★ | ★ | ★ | 8 |
| R Langton 2022 | ★ | ★ | × | ★ | ★★ | × | ★ | ★ | 7 |
| Sydsjö 2020 | ★ | ★ | ★ | ★ | ★★ | ★ | ★ | ★ | 9 |
| S. Sadrzadeh 2017 | ★ | × | ★ | - | ★ | × | ★ | × | 4 |
| K Bjelland 2020 | ★ | ★ | × | ★ | ★★ | × | ★ | ★ | 7 |

Notes: “★” represents 1 point, “×” represents 0 point, and “—” represents uncertain points.

Abbreviation: NOS, Newcastle–Ottawa Scale.

**Supplementary table 4.** Reasons for study exclusion.

| **Study** | **Year** | **Reason for exclusion** | **Number of studies** |
| --- | --- | --- | --- |
| Treloar SA, et al. (1) | 2000 | Wrong outcome | 9 |
| R Hardy, et al. (2) | 2002 |  |  |
| D E Yarbrough, et al. (3) | 1998 |  |  |
| M Gao, et al. (4) | 2018 |  |  |
| Sorina Grisaru-Granovsky, et al. (5) | 2015 |  |  |
| Gita Mishra, et al. (6) | 2007 |  |  |
| Fu-Sheng Chou, et al. (7) | 2020 |  |  |
| Jeffrey R Thompson, et al. (8) | 2003 |  |  |
| L. V. Tkachenko, et al. (9) | 2023 |  |  |
| Mandy Goldberg, et al. (10) | 2020 | No data for birth weight | 2 |
| F Yarde, et al. (11) | 2013 |  |  |
| Anne Z. Steiner, et al. (12) | 2010 | No data for [premature ovarian failure](javascript:;) | 2 |
| J L Cresswell, et al. (13) | 1997 |  |  |
| Barbara T, et al. (14) | 2014 | Wrong study type | 4 |
| Rinky Giri, et al. (15) | 2020 |  |  |
| S Sadrzadeh, et al.（16） | 2017 |  |  |
| Gita D Mishra, et al.（17） | 2019 |  |  |
| Marzie Reisi, et al. (18) | 2001 | Non-English language | 1 |

**References**

1. Susan A.Treloar and others, Birth weight and age at menopause in Australian female twin pairs: exploration of the fetal origin hypothesis, Human Reproduction, Volume 15, Issue 1, January 2000, Pages 55–59, <https://doi.org/10.1093/humrep/15.1.55>.

2. Hardy R, Kuh D. Does early growth influence timing of the menopause? Evidence from a British birth cohort. Hum Reprod. 2002 Sep;17(9):2474-9. doi: 10.1093/humrep/17.9.2474. PMID: 12202444.

3. Yarbrough DE, Barrett-Connor E, Kritz-Silverstein D, Wingard DL. Birth weight, adult weight, and girth as predictors of the metabolic syndrome in postmenopausal women: the Rancho Bernardo Study. Diabetes Care. 1998 Oct;21(10):1652-8. doi: 10.2337/diacare.21.10.1652. PMID: 9773725.

4. Gao M, Goodman A, Mishra G, Koupil I. Associations of birth characteristics with perimenopausal disorders: a prospective cohort study. J Dev Orig Health Dis. 2019 Apr;10(2):246-252. doi: 10.1017/S204017441800065X. Epub 2018 Oct 9. PMID: 30296955.

5. Grisaru-Granovsky S, Gordon ES, Haklai Z, Schimmel MS, Drukker L, Samueloff A, Keinan-Boker L. Delivery of a very low birth weight infant and increased maternal risk of cancer and death: a population study with 16 years of follow-up. Cancer Causes Control. 2015 Nov;26(11):1593-601. doi: 10.1007/s10552-015-0653-x. Epub 2015 Aug 28. PMID: 26316180.

6. Mishra G, Hardy R, Kuh D. Are the effects of risk factors for timing of menopause modified by age? Results from a British birth cohort study. Menopause. 2007 Jul-Aug;14(4):717-24. doi: 10.1097/GME.0b013e31802f3156. PMID: 17279060.

7. Chou FS, Yeh HW, Chen CY, Lee GT, Parrish MR, Omede M, Pandey V. Exposure to placental insufficiency alters postnatal growth trajectory in extremely low birth weight infants. J Dev Orig Health Dis. 2020 Aug;11(4):384-391. doi: 10.1017/S2040174419000564. Epub 2019 Oct 4. PMID: 31581967.

8. Thompson JR, Carter RL, Edwards AR, Roth J, Ariet M, Ross NL, Resnick MB. A population-based study of the effects of birth weight on early developmental delay or disability in children. Am J Perinatol. 2003 Aug;20(6):321-32. doi: 10.1055/s-2003-42773. PMID: 14528402.

9. Tkachenko L.V., Gritsenko I.A., Tikhaeva K.Yu., Sviridova N.I., Gavrilova I.S., Dolgova V.A. Assessing an ovarian reserve and risk factors for premature ovarian failure as part of pre-abortion counseling for women under 40 planning to terminate own first pregnancy. Obstetrics, Gynecology and Reproduction. 2023;17(2):244-251. (In Russ.) https://doi.org/10.17749/2313-7347/ob.gyn.rep.2023.369

10. Goldberg M, Tawfik H, Kline J, Michels KB, Wei Y, Cirillo P, Cohn BA, Terry MB. Body size at birth, early-life growth and the timing of the menopausal transition and natural menopause. Reprod Toxicol. 2020 Mar;92:91-97. doi: 10.1016/j.reprotox.2019.02.013 . Epub 2019 Mar 1. PMID: 30831215.

11. Yarde F, Broekmans FJ, van der Pal-de Bruin KM, Schönbeck Y, te Velde ER, Stein AD, Lumey LH. Prenatal famine, birthweight, reproductive performance and age at menopause: the Dutch hunger winter families study. Hum Reprod. 2013 Dec;28(12):3328-36. doi: 10.1093/humrep/det331. Epub 2013 Aug 21. PMID: 23966246; PMCID: PMC3895982.

12. Steiner AZ, D'Aloisio AA, DeRoo LA, Sandler DP, Baird DD. Association of intrauterine and early-life exposures with age at menopause in the Sister Study. Am J Epidemiol. 2010 Jul 15;172(2):140-8. doi: 10.1093/aje/kwq092. Epub 2010 Jun 9. PMID: 20534821; PMCID: PMC2915484.

13. Cresswell JL, Egger P, Fall CH, Osmond C, Fraser RB, Barker DJ. Is the age of menopause determined in-utero? Early Hum Dev. 1997 Sep 19;49(2):143-8. doi: 10.1016/s0378-3782(97)00028-5. PMID: 9226121.

14. Alexander BT, Henry Dasinger J, Intapad S. Effect of low birth weight on women's health. Clin Ther. 2014 Dec 1;36(12):1913-1923. doi: 10.1016/j.clinthera.2014.06.026. Epub 2014 Jul 23. PMID: 25064626; PMCID: PMC4268017.

15. Giri R, Vincent AJ. Prevalence and Risk Factors of Premature Ovarian Insufficiency/Early Menopause. Semin Reprod Med. 2020 Sep;38(4-05):237-246. doi: 10.1055/s-0040-1722317. Epub 2021 Jan 12. PMID: 33434933.

16. Sadrzadeh S, Verschuuren M, Schoonmade LJ, Lambalk CB, Painter RC. The effect of adverse intrauterine conditions, early childhood growth and famine exposure on age at menopause: a systematic review. J Dev Orig Health Dis. 2018 Apr;9(2):127-136. doi: 10.1017/S2040174417000952. Epub 2017 Dec 4. PMID: 29198238.

17. Mishra GD, Chung HF, Cano A, Chedraui P, Goulis DG, Lopes P, Mueck A, Rees M, Senturk LM, Simoncini T, Stevenson JC, Stute P, Tuomikoski P, Lambrinoudaki I. EMAS position statement: Predictors of premature and early natural menopause. Maturitas. 2019 May;123:82-88. doi: 10.1016/j.maturitas.2019.03.008. Epub 2019 Mar 13. PMID: 31027683.

18. Reisi, M., Asadi, L. The relationship between fetal environment and menopausal age: A narrative review study. The Iranian Journal of Obstetrics, Gynecology and Infertility, 2021; 24(8): 84-94. doi: 10.22038/ijogi.2021.19071.
